# Very Sensitive

**Published:** 1856-09

**Authors:** 


### EDITORIAL.

*Very Sensitive.*

Our readers will remember that we published the entire proceedings of the American Medical Association in the June number of the *Journal*, accompanied by an editorial paragraph commendatory of everything except the *time* selected by the Committee of Arrangements for a Steamboat Excursion. We made no objection to the excursion itself, but only to the *time* chosen for taking it, viz.: the middle of the session and the best working day in it. This simple objection, which contained not a word disrespectful to the Committee of Arrangements, has called forth a formal response in the August number of the *Peninsular Journal.* In that response, the writer, after expressing "the gratification they (the Committee) have derived from the general manifestation of approval of those arrangements which have reached them from all parts of the Union since the return of the delegates to their homes," says, in reference to the boat-ride and our objections, as follows:—

"We cannot say that this departure from the usage of the Association was universally approved. The gentlemen composing it have at least quietly acquiesced in the arrangement. The complacency of the Committee has only been disturbed by a single voice of disapprobation coming from the editor of the *North-Western Med. and Surg. Journal.*

"As the business of the session was completed before the members of the Association dispersed, we are unable to surmise what loss was sustained by the suspension of labor on Thursday afternoon, unless it was the opportunity of listening to the annual speech of Dr. Davis, of Chicago. And as that gentleman has caused his propensities for water to be widely known, we are equally at a loss to comprehend the reasons for his preference of the evening parties where the aroma of a Roman punch bowl constituted one of the attractions, over an aquatic excursion conducted on temperance principles, unless it be a constitutional tendency to find fault, or a cultivated design to disapprove of whatever has not germinated in Binghamton and been transplanted to Chicago."

This paragraph, besides manifesting a morbid sensibility to criticism, contains mistakes, misrepresentations, and personal insinuations quite characteristic of the source from which it emanates. First, the "suspension of labor" for the excursion took place on *Wednesday*, the second day of the session, and not on "*Thursday afternoon*," as stated by "*one of the Committee.*" Second, we are represented as having expressed a "preference of evening parties, where the aroma of the Roman punch bowl constituted one of the attractions, over an aquatic excursion conducted on temperance principles." We neither expressed nor implied any such preference, and, if the "*Aroma of the Roman Punch Bowl*" constituted one of the *attractions* at the evening parties, the more shame to those who gave them, among whom was doubtless the very individual who penned the article and signed himself "One of the Committee." The personal insinuations about the "annual speech of Dr. Davis"—

his "constitutional tendency to find fault," &c., serve no other purpose than to indicate the feeling which animated the writer. Coming, as they do, doubtless, from one who might just as well sign himself, "One of the Editors of the *Peninsular Journal*, or President of the American Medical Association, as "One of the Committee," they will be duly appreciated.

